# *Piper nigrum* Extract Inhibits the Growth of Human Colorectal Cancer HT-29 Cells by Inducing p53-Mediated Apoptosis

**DOI:** 10.3390/ph16091325

**Published:** 2023-09-19

**Authors:** Rui Wu, Jiajia Zhao, Panhong Wei, Minghai Tang, Ziyan Ma, Yunyan Zhao, Leilei Du, Li Wan

**Affiliations:** 1 State Key Laboratory of Southwestern Chinese Medicine Resources, School of Pharmacy, Chengdu University of Traditional Chinese Medicine, Chengdu 611137, China; wurui2298@163.com (R.W.); zjj11242020@163.com (J.Z.); wph0816@163.com (P.W.); yunyan_zhao2022@163.com (Y.Z.); duleilei@cdutcm.edu.cn (L.D.); 2 State Key Laboratory of Biotherapy and Cancer Center, National Clinical Research Center for Geriatrics, West China Hospital of Sichuan University, Chengdu 610041, China; tangminghai123@sina.cn (M.T.); maziyan@stu.scu.edu.cn (Z.M.)

**Keywords:** *Piper nigrum*, colorectal cancer, HT-29 cells, apoptosis, p53

## Abstract

Colorectal cancer (CRC) is a prevalent malignancy of the digestive tract with the second highest mortality rate globally. *Piper nigrum* is a widely used traditional medicinal plant, exhibiting antitumor activity against various tumor cells. At present, research on the effect of *Piper nigrum* on CRC is limited to in vitro cytotoxicity, lacking comprehensive mechanism investigations. This study aimed to explore the inhibitory effect and mechanism of *Piper nigrum* extract (PNE) on HT-29 cells. Firstly, we identified the chemical components of PNE. Then, MTT assay, colony formation assay, JC-1 staining, and flow cytometry were used to analyze the effect of PNE on HT-29 cells in vitro. A xenograft model, histopathological examination, immunohistochemistry, and western blot were used to evaluate the tumor growth inhibitory activity and mechanism of PNE in vivo. The results indicated that PNE could inhibit cell proliferation and colony formation, reduce mitochondrial membrane potential, induce cell apoptosis in vitro, and inhibit tumor growth in vivo. Furthermore, PNE could regulate p53 and its downstream proteins, and subsequently activate the caspase-3 pathway. In summary, PNE probably induced apoptosis of HT-29 cells through the mitochondrial pathway mediated by p53. All these results suggested that PNE might be a potential natural-origin anti-CRC drug candidate.

## 1. Introduction

Colorectal cancer (CRC) stands as the prevailing malignancy within the digestive tract. According to the most recent worldwide cancer statistics from 2020, CRC has become the third most common type of cancer globally, with its mortality rate ranking second [1]. Surgery and chemotherapy are the main clinical treatments for CRC currently. However, because of the occurrence of specific adverse effects and the emergence of drug resistance, it is necessary to research novel drugs to treat CRC more effectively [2,3]. Nowadays, natural products have become important sources of new anticancer drugs, and the scientific exploration of them originated in the 1950s. At present, approximately 50% of the anticancer medications employed in clinical practice originate either directly or indirectly from natural sources. These sources encompass compounds such as analogs of vinca alkaloids, derivatives of camptothecin, derivatives of podophyllotoxin, and semi-synthetic analogs of taxol [4]. An increasing number of studies have found that various natural products such as alkaloids, polysaccharides, and terpenoids can exert anti-CRC effects on cell proliferation, metastasis, and apoptosis. This indicates that natural-origin drugs have broad application prospects for the treatment of CRC.

*Piper nigrum* is a medicinal plant from the family *Piperaceae*, which was also known as the “king of spices”. *Piper nigrum* is used to treat fever, asthma, gastrointestinal diseases, and cancer in the traditional medicine of China, Thailand, and Ayurveda [5]. The major active compounds in *Piper nigrum* are alkaloids, such as piperine, piperolein B, and piperyline, while coumarins, lignans, and organic acids are other important compounds [6]. According to modern medical research, *Piper nigrum* extract (PNE) and its compounds show biological activities such as antitumor, anti-inflammatory, and antioxidant properties, among which the antitumor effect is currently a hot topic in the pharmacological research of *Piper nigrum* [5]. Piperine, as the most important active compound in *Piper nigrum*, has been proven to inhibit the growth of tumor cells by blocking the cell cycle, inducing apoptosis, repressing cell migration, etc. [7,8]. In addition, piperine-free *Piper nigrum* extract has also been proven to have anti-breast cancer activity in vivo and in vitro, as well as displayed cytotoxicity against colorectal and lung cancer cells [9,10]. (-)-Kusunokinin and piperloguminine, obtained from *Piper nigrum*, exerted cytotoxicity against breast cancer cells through G2/M phase arrest and regulation of extrinsic pathways such as Bcl-2 and p53 [11]. Pillitorine, another constituent derived from *Piper nigrum*, demonstrated potent cytotoxic effects when tested against HL60 and MCT-7 cell lines [12].

The p53 gene is a tumor suppressor gene that can induce cell apoptosis through the mitochondrial pathway and the “death receptor” pathway [13]. Mutations in the p53 gene are strongly correlated to the occurrence and development of tumors. According to reports, p53 has a 60% mutation frequency in CRC and is considered one of the main etiological mechanisms of CRC [14]. Therefore, finding natural-origin drugs targeting p53 may be a prospective direction for CRC treatment.

A study investigated the cytotoxicity effect of *Piper nigrum* ethanolic extract on CRC cell lines in vitro. The results indicated the potential of *Piper nigrum* ethanolic extract as a new anti-CRC drug [15]. Nevertheless, there is currently a lack of in vivo and mechanism research on the anti-CRC effect of *Piper nigrum* [6,16]. Consequently, the objective of this study was to investigate the inhibitory impact of PNE on HT-29 cells both in vitro and in vivo, and also to investigate the effect of PNE on p53-mediated mitochondrial apoptosis. The results serve as a preclinical research basis for the further development and utilization of PNE as an anticancer drug.

## 2. Results

### 2.1. Qualitative Analysis of PNE

The obtained PNE was a brown lyophilized powder with an extraction yield of 15.68%. UPLC-Q-Exactive Plus MS was used to detect the components in PNE. A total of 97 compounds were screened and identified (Table S1), including alkaloids, coumarins, phenylpropanoids, organic acids, etc. Piperine was the most abundant alkaloid, with a relative content of 23.05% of all peaks. There were 13 compounds with pharmacological activity and a relatively high content (Figure S1, Table 1). The vast majority were amide alkaloids in addition to one phenylpropanoid compound, propafenone. Among them, piperine, piperolein B, pellitorine, N-trans-feruloyltyramine, trigonelline, betaine, oleamide, and 4,5-dihydropiperlonguminine have all been reported to have antitumor activity.

### 2.2. PNE Inhibited the Proliferation of HT-29 Cells

**Figure 1:**
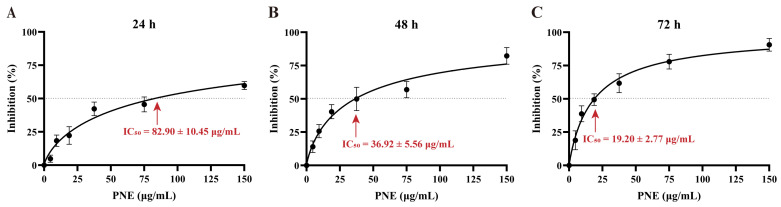
Proliferation inhibition rates and IC_50_ values. (**A**) PNE treated, 24 h, (**B**) PNE treated, 48 h, (**C**) PNE treated, 72 h. Data are expressed as mean ± SD (*n* = 6).

Cell proliferation inhibition was determined using the MTT assay. As depicted in Figure 1, the inhibitory effects exhibited an escalation in line with both dosage and duration. The IC_50_ values of PNE at 24 h, 48 h, and 72 h were 82.90 ± 10.45 μg/mL, 36.92 ± 5.56 μg/mL, and 19.20 ± 2.77 μg/mL, respectively.

### 2.3. PNE Inhibited Colony Formation in HT-29 Cells

**Figure 2:**
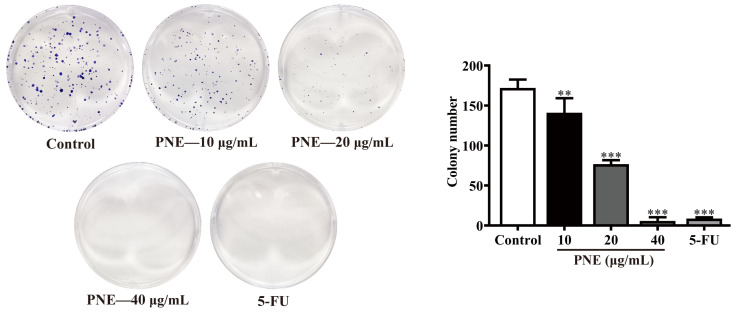
The effect of PNE on the colony formation ability of HT-29 cells. The results are presented as the mean ± SD (*n* = 3). ** *p* < 0.01, *** *p* < 0.001 compared to control group.

The proliferative capacity of individual cells was assessed using the colony formation assay. The results shown in Figure 2 indicate that PNE could significantly reduce the number of colonies in HT-29 cells in a dose-dependent manner (*p* < 0.01).

### 2.4. PNE Induced Apoptosis in HT-29 Cells

**Figure 3:**
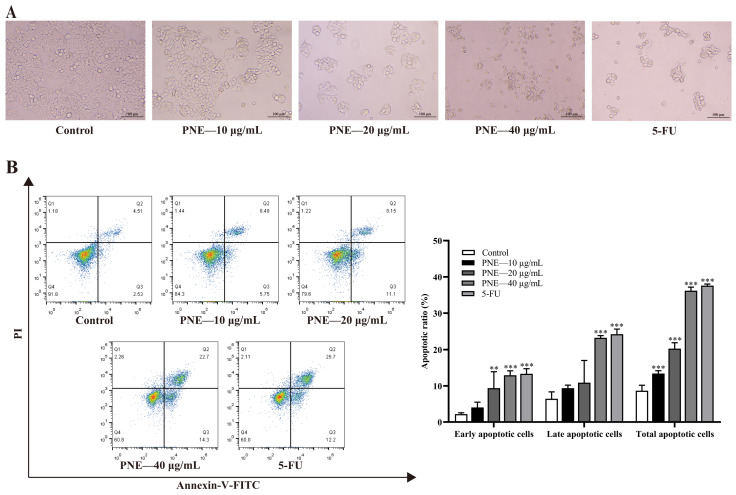
The apoptosis induction effects of PNE on HT-29 cells. (**A**) Effect of PNE on cell morphology (×200). (**B**) Flow cytometry analysis of apoptosis in HT-29 cells. Statistical analysis was conducted on the early apoptotic ratio (lower right quadrant), late apoptotic ratio (upper right quadrant), and total apoptotic ratio. The results are presented as the mean ± SD (*n* = 3). ** *p* < 0.01, *** *p* < 0.001 compared to the control group.

Cell apoptosis was determined via annexin V–FITC/PI staining. As shown in Figure 3A, the cells gradually shrank and became rounder, exhibiting apoptotic morphology after treatment with PNE. The flow cytometry analysis results are shown in Figure 3B. PNE treatment could induce early and late apoptosis of HT-29 cells to varying degrees, and increase the total apoptosis ratio in a dose-dependent manner (*p* < 0.001). The results indicated that PNE could effectively promote the apoptosis of HT-29 cells.

### 2.5. PNE Reduced the Mitochondrial Membrane Potential (MMP) of HT-29 Cells

**Figure 4:**
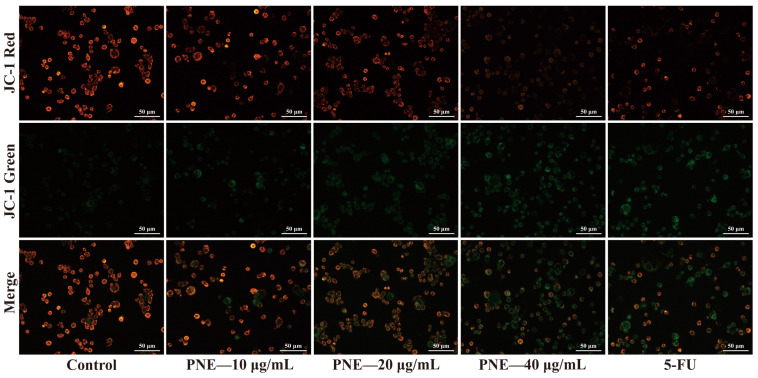
MMP disruption in HT-29 cells imaged using an inverted fluorescent microscope (×200).

To explore the apoptosis pathway, a JC-1 staining assay was employed to identify the alterations in MMP. As shown in Figure 4, the cells in the control group showed bright red fluorescence, while the green fluorescence was weak. With the increase in the PNE concentration, the red fluorescence of cells in the PNE-treated group gradually weakened and the green fluorescence gradually increased. The results showed that PNE could induce MMP dissipation.

### 2.6. PNE Inhibited Tumor Growth In Vivo

**Figure 5:**
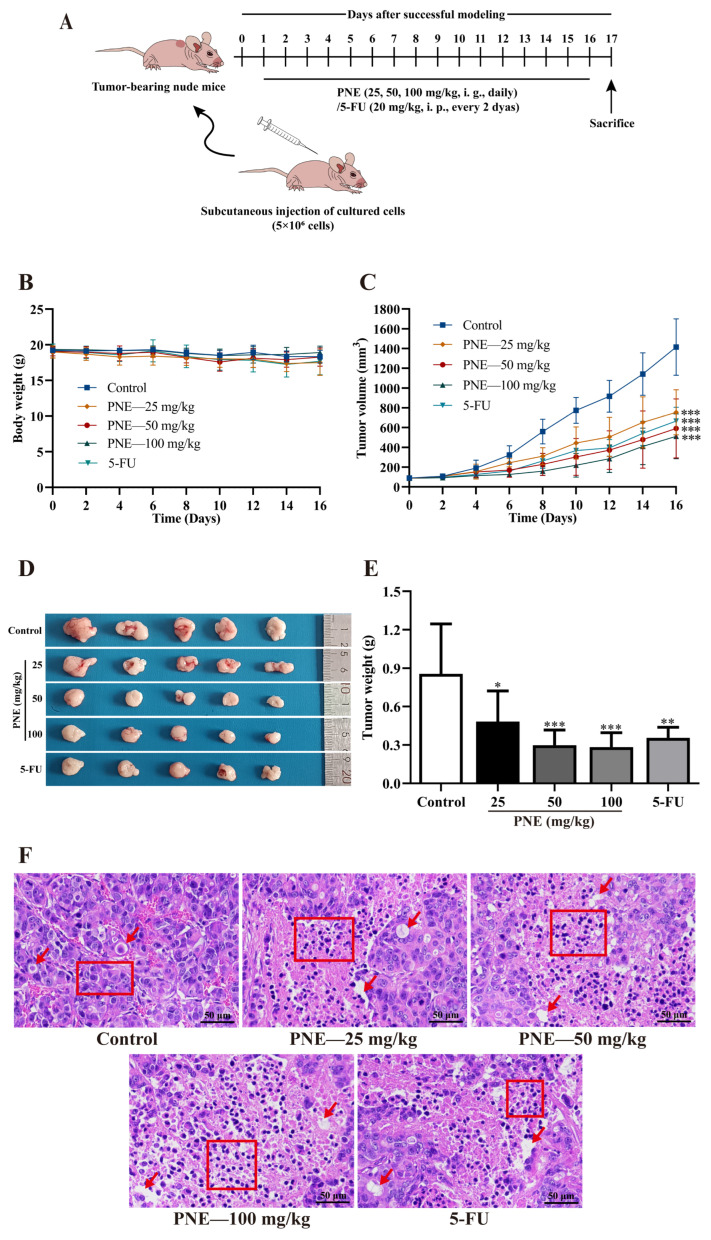
The inhibitory activity of PNE in an HT-29 nude mouse xenograft model. (**A**) Schematic representation of the establishment and treatment of xenograft model; i. g. means intragastric administration, i.p. means intraperitoneal administration. (**B**) Growth curves of the body weight. (**C**) Growth curves of the tumor volume. (**D**) Images of the tumors. (**E**) Tumor weight. (**F**) Histopathological examination of the tumor tissues (×200). The results are presented as the mean ± SD (*n* = 5). * *p* < 0.05, ** *p* < 0.01, *** *p* < 0.001 compared to the control group.

In order to evaluate the inhibitory effect of PNE on HT-29 cells in vivo, an HT-29 nude mouse xenograft model was established in this study (Figure 5A). As shown in Figure 5, over the course of the treatment period, the average body weight of mice in each group displayed no significant alteration (*p* > 0.05). After 16 days of treatment, the tumor volumes and tumor weights of mice within the treatment groups exhibited marked reductions compared to those in the control group (*p* < 0.05). The tumor inhibition rates of PNE 25, 50, and 100 mg/kg groups, and the 5-fluorouracil (5-FU) group were 43.56%, 65.10%, 66.88%, and 58.38%, respectively. The above findings indicated a significant inhibition of tumor growth by PNE, accompanied by low toxicity and few side effects at experimental doses.

Furthermore, in the histopathological examination of the tumors, it was observed that, in comparison to the control group, cells within the treatment groups exhibited disordered arrangement. Moreover, an increase in the cytoplasmic region was noted, accompanied by varying degrees of nuclear pyknosis (as indicated by the red boxes in Figure 5F). Additionally, partial cells displayed necrosis and dissolution, resulting in void formation (as marked by the red arrows in Figure 5F). These results indicated that PNE could cause apoptosis and necrosis of HT-29 tumor cells in vivo.

### 2.7. PNE Regulated the Expression of p53 and Its Downstream Target Proteins

**Figure 6:**
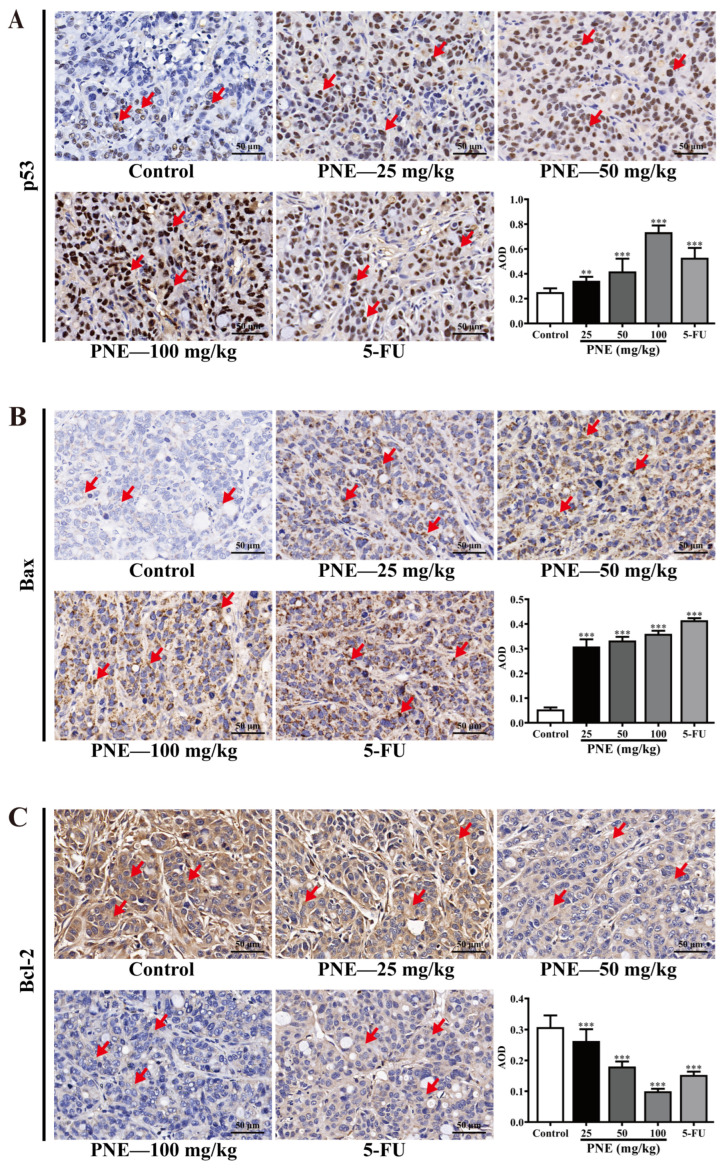
IHC images and expression levels of p53, Bax, and Bcl-2 in tumor tissues (×400). (**A**) The expression of p53 detected using IHC. (**B**) The expression of Bax detected using IHC. (**C**) The expression of Bcl-2 detected using IHC. The IHC images were analyzed using ImageJ software. The results are presented as the mean ± SD (*n* = 9). ** *p* < 0.01, *** *p* < 0.001 compared to the control group.

To further explore the inhibitory mechanism of PNE on HT-29 cells, the in situ expressions of p53, Bax, and Bcl-2 proteins in mice tumor tissues were analyzed using immunohistochemistry (IHC). As shown in Figure 6, the p53 protein was expressed in the nucleus, and the Bax and Bcl-2 proteins were expressed in the cytoplasm (as marked by the red arrows). The results demonstrated a dose-dependent increase in the expression of the p53 and Bax proteins in the PNE groups (*p* < 0.05). On the contrary, the expression of the Bcl-2 protein in the PNE groups exhibited a significant decrease in a dose-dependent manner (*p* < 0.05).

**Figure 7:**
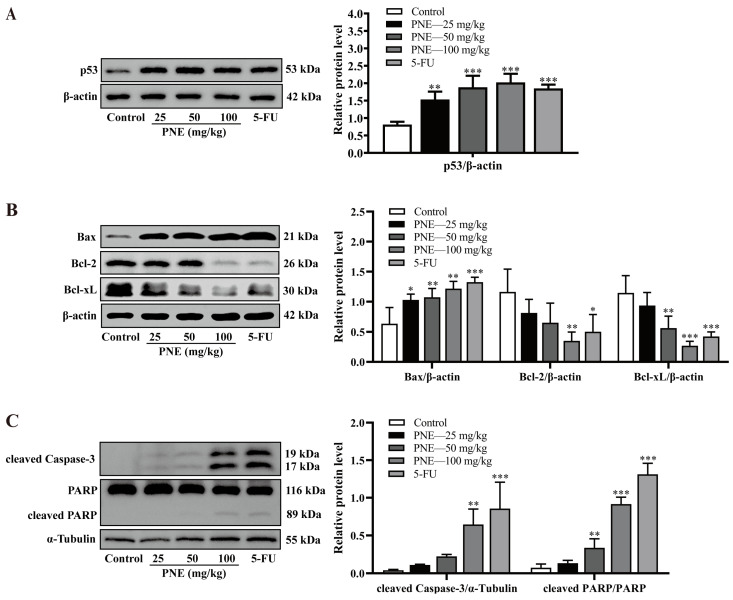
Expression of p53 and its downstream target proteins in tumor tissues detected using WB. (**A**) The expression of p53 protein using WB. (**B**) The expression of Bax, Bcl-2, and Bcl-xL proteins detected using WB. (**C**) The expression of cleaved caspase-3, PARP, and cleaved PARP proteins detected using WB. The relative protein levels were processed using ImageJ software. The results are presented as the mean ± SD (*n* = 3). * *p* < 0.05, ** *p* < 0.01, *** *p* < 0.001 compared to the control group.

Furthermore, this study also used western blot (WB) assays to semi-quantitatively measure the expression of p53 and its downstream apoptosis-related proteins in tumor tissues. As shown in Figure 7A,B, PNE could significantly upregulate the expression levels of p53 and Bax proteins dose-dependently (*p* < 0.05), while decreasing the Bcl-2 and Bcl-xL protein levels. In addition, PNE could also increase the expression of cleaved caspase-3 protein, and promote the cleavage of PARP into cleaved PARP (Figure 7C).

## 3. Discussion

*Piper nigrum* is one of the most popular spices in the world and a widely used traditional natural medicine [5]. Analyzing the chemical composition of *Piper nigrum* helps to understand its pharmacological mechanisms. As the identification results showed in this study, alkaloids were the main components of PNE, with piperine being the most abundant alkaloid. Current research has confirmed the in vitro inhibitory effect of piperine on HT-29 cells. For instance, Huo et al. [31] found that piperine inhibited HT-29 cell proliferation in vitro in a dose-dependent manner. A study by Shaheer et al. [32] indicated that piperine pretreatment enhanced radio-sensitization in HT-29 cells by promoting apoptosis through a mitochondria-dependent pathway. These studies about piperine provide evidence for the anti-CRC effects of PNE. And in addition to piperine, a variety of other alkaloids with anti-tumor and other pharmacological activities in PNE were also found. The above results suggested that the antitumor effects of *Piper nigrum* may be achieved by the synergy of multiple active components through complementary mechanisms [33].

After the characterization of the chemical components in PNE, we investigated the anti-CRC effects of PNE in vitro. The results of the MTT assay and colony formation assay suggested that PNE could significantly inhibit the viability and single-cell proliferation of HT-29 cells [14]. Apoptosis, a programmed cell death process, stands as one of the inherent checks and balances of the cell cycle. It can promptly eliminate non-functional, harmful, and abnormal cells. The blocking of apoptosis is one of the characteristics of tumor cells. Thus, treatment strategies that target the regulation of apoptosis pathways are essential directions for cancer treatment [13]. *Piper nigrum* and its components have the effect of inducing apoptosis in different types of tumor cells. For example, it had been reported that *Piper nigrum* ethanolic extract, enriched with piperamides, could hinder the growth of Ehrlich ascites carcinoma by inducing cell cycle arrest and promoting apoptosis [16]. Piperine could promote endoplasmic reticulum stress-associated apoptosis of HT-29 cells [7]. (-)-Kusunokinin and piperloguminine, derived from *Piper nigrum*, induced cell apoptosis in MCF-7 and MDA-MB-468 cells [11]. In this study, the results of the annexin V–FITC/PI staining assay indicated that PNE had the capability to induce both early and late apoptosis in HT-29 cells. Apoptosis can be triggered through the intrinsic pathway, mediated by mitochondria, and/or the extrinsic pathway, mediated by death receptors [33]. The collapse of MMP is one of the signs of activation of the mitochondrial death pathway [34]. The JC-1 method is commonly used to detect MMP, where JC-1 is used as a fluorescent probe to reveal the change in MMP by the transformation of the fluorescent color [35]. In this study, the JC-1 experiment showed that PNE could reduce the MMP of HT-29 cells. All of the above suggested that PNE may induce apoptosis in HT-29 cells through the mitochondrial-mediated pathway, thereby inhibiting cell proliferation.

Cell-line-derived tumor xenograft models are widely used for screening antitumor drugs in vivo [36]. This study established an HT-29 nude mouse xenograft model for in vivo antitumor studies. The results indicated that PNE could significantly inhibit tumor growth with minimal toxic and side effects. When formulating the treatment plan, considering that PNE was a crude extract with complex components, we chose the safer intragastric administration method. According to a previous paper, the use of oral 5-FU had been abandoned early owing to its unpredictable gastrointestinal absorption and marked variation in pharmacokinetics [37]. Meanwhile, due to the strong toxic side effects of 5-FU as a chemotherapy drug, we ultimately chose intraperitoneal injection of 5-FU every 2 days for the positive control group. Apoptosis is accompanied by morphological changes [38]. The tumor histopathological examination results were consistent with the morphological changes in cells after in vitro treatments, confirming the proapoptotic effects of PNE on HT-29 cells in vivo.

As previously mentioned, evading apoptosis is pivotal in carcinogenesis, involving three key mechanisms: (1) disrupted balance between pro-apoptotic and antiapoptotic proteins, (2) reduced caspase activity, and (3) impaired death receptor signaling [13]. Apoptosis-related proteins include the p53 protein, the Bcl-2 family proteins, and apoptosis inhibitors.

Encoded by the TP53 tumor suppressor gene, the p53 protein stands is among the earliest identified tumor suppressor proteins. It plays a crucial role in regulating multiple cellular processes such as apoptosis, cell cycle arrest, and DNA repair, by controlling the expression of numerous targets. Due to its functions, it is often referred to as the “guardian of the genome” [13]. The p53 mutations can result in the loss or alteration in the binding activity between p53 and its downstream targets, thereby inducing abnormal cell proliferation, subsequently leading to malignant cellular transformation [39]. Currently, p53 has been found to mutate or lose in over 50% of all human cancer cases. For instance, p53 mutation frequency is 60% in colon cancer, 70% in lung cancer, and 45% in stomach cancer [14]. Enhancing wild-type p53 function or stabilizing p53 to treat cancer are currently effective strategies [39]. The Bcl-2 proteins family includes both pro-apoptotic and antiapoptotic members, mainly regulating cell apoptosis through the mitochondrial pathway. Pro-apoptotic proteins such as Bax and Bak can activate downstream caspases by releasing cytochrome C from mitochondria upon receipt of signals, leading to cell apoptosis. Anti-apoptotic proteins like Bcl-2, Bcl-X (Bcl-xS and Bcl-xL), and Bcl-w inhibit apoptosis through heterodimer formation with pro-apoptotic proteins [40].

The Bcl-2 family proteins are closely associated with p53 function. For example, Bax, as the first discovered p53-regulated pro-apoptotic Bcl-2 family protein, can participate in the death response as an indirect target of p53 through Puma. In mitochondria, p53 forms an inhibitory complex by binding to Bcl-xL and Bcl-2 proteins directly to displace Bax, thereby facilitating Bax-dependent mitochondrial apoptosis [39].

Caspase family proteins are crucial for initiating and executing apoptosis. caspase-3 protein serves as the downstream target in the mitochondrial apoptosis signaling pathway influenced by Bcl-2 family proteins. Poly (ADP-ribose) polymerase (PARP) stands as a significant substrate for caspase-3 cleavage, aiding cells in maintaining survival ability. After activation, caspase-3 undergoes cleavage. Cleaved caspase-3 cleaves PARP into cleaved-PARP, which promotes cellular disassembly and has been widely utilized as a hallmark of apoptosis [41].

This study used IHC and WB methods to detect the expression of p53 and its downstream proteins. The results revealed the pro-apoptotic mechanisms of PNE, which might be that the p53 protein was activated by PNE, then bound to Bcl-2 and Bcl-xL proteins, thus replacing Bax and induced permeabilization of the outer mitochondrial membrane, then caspase-3 was activated so that PARP was cleaved. Ultimately, the cell apoptosis through the mitochondrial pathway was activated.

## 4. Materials and Methods

### 4.1. Chemical Reagent

Chromatographic grade methanol, analytical grade ethanol, and 5-FU (≥99% HPLC purity) were procured from Sigma-Aldrich (St. Louis, MO, USA). Formic acid (chromatographic grade) was sourced from Chron Chemicals Company (Chengdu, China). MTT was bought from Saiguo Biotech Co., Ltd. (Guangzhou, China). RPMI-1640 medium, trypsin 0.25% solution, and penicillin–streptomycin solution were bought from Hyclone (South Logan, UT, USA). FBS was obtained from Yeasen Biotech Co., Ltd. (Shanghai, China). The annexin V–FITC/PI apoptosis kit was bought from MultiSciences Biotech Co., Ltd. (Hangzhou, China). Crystal violet staining solution and the mitochondrial membrane potential assay kit with JC-1 were acquired from Beyotime Biotech, Inc. (Shanghai, China). p53, Bax, Bcl-2, and Bcl-xL rabbit monoclonal antibodies were bought from Zen Bioscience Co., Ltd. (Chengdu, China). Cleaved caspase-3 and PARP rabbit monoclonal antibodies were sourced from Cell Signaling Technology (Beverly, MA, USA). β-actin rabbit polyclonal antibody, goat anti-mouse and goat anti-rabbit IgG (H + L) HRP secondary antibodies were obtained from Abcam (Cambridge, MA, USA). α-Tubulin mouse monoclonal antibody was purchased from Proteintech Group, Inc. (Wuhan, China).

### 4.2. Plant Material

The dried unripe fruits of *Piper nigrum* (*Piperaceae*) were purchased from the Chengdu Hehuachi Chinese Herbal Medicine Market and authenticated by Professor Zhuyun Yan from Chengdu University of Traditional Chinese Medicine, Sichuan, China. The voucher specimens (No. 20210910) were placed in a glass desiccator containing desiccants and stored at room temperature in the College of Pharmacy, Chengdu University of Traditional Chinese Medicine.

### 4.3. Preparation and UPLC-Q-Exactive Plus MS analysis of PNE

The fruits of *Piper nigrum* were crushed into coarse powder and extracted three times using a heat reflux method, with 8 times, 6 times, and 6 times the volume of 70% ethanol for the first, second, and third extraction step, respectively, for 1.5 h. Afterwards, the filtrates from the three extractions were combined, concentrated under reduced pressure at 70–80 °C, and subsequently freeze–dried at −80 °C at a pressure of less than 20 Pa to yield PNE. A small amount of PNE was dissolved in methanol and characterized using UPLC-Q-Exactive Plus MS (Thermo Fisher Scientific; Waltham, MA, USA). Chromatographic separations were performed using a Hypersil GOLD C18 column (100 mm × 2.1 mm, 1.9 μm; Thermo Fisher Scientific; Waltham, MA, USA) at 35 °C. The mobile phase consisted of water with 0.1% formic acid (A) and methanol (B). The following gradient procedure was employed: 0–3 min, 10–70% B; 3–8 min, 70% B; 8–15 min, 70–90% B; 15–20 min, 90–95% B. The mass spectrum was set to full MS-ddMS2 mode and run over a scan range of *m*/*z* 100–1500 Da. The obtained mass spectrum peaks were matched with the online databases mzVault and mzCloud and analyzed using Compound Discoverer software (version 3.0; Waltham, MA, USA) to screen compounds with matching degrees >80%. Subsequently, the compounds screened were further identified by integrating the information from the secondary fragment ions in the MS/MS spectrum.

### 4.4. Cell Culture

The human CRC cell line (HT-29), acquired from the American Type Culture Collection (ATCC; Manassas, VA, USA), was cultured in RPMI-1640 medium containing 10% (*v*/*v*) FBS and 1% (*v*/*v*) penicillin–streptomycin solution at 37 °C with 5% CO_2_.

### 4.5. Cell Proliferation Inhibition Assay

HT-29 cells in the logarithmic growth phase were seeded into 96-well microplates at a density of 3 × 10^3^ cells/well, and treated with a series of gradient concentrations of PNE (0, 4.6875, 9.375, 18.75, 37.5, 75, and 150 μg/mL) for 24, 48, and 72 h. After a 3 h incubation with MTT (5mg/mL), formazan crystals were solubilized using DMSO. Subsequently, the optical density (OD) was measured at 570 nm using a microplate reader (Bio-Rad; Hercules, CA, USA). The inhibition of cell proliferation was expressed as the relative inhibition rate: Inhibition% = [1 − (ODtest − ODblank)/(ODcontrol − ODblank)] × 100%. GraphPad Prism (version 8.0.2, La Jolla, CA, USA) was utilized to fit the concentration–effect curve and determine the IC_50_ value.

### 4.6. Colony Formation Assay

HT-29 cells were plated in 6-well microplates at 500 cells/well and treated with PNE (10, 20, and 40 μg/mL) and 5-FU (20 μg/mL) for 12 days (5-FU, an important chemotherapeutic drug for CRC [42], was used as the positive control), with the drug-containing fresh medium replaced every 3 days. The cells were washed twice with PBS, fixed using 4% paraformaldehyde (1.5 mL/well) for 30 min, and then stained with crystal violet staining solution (1.5 mL/well) for 15 min. Finally, pictures were captured using a camera after washing the cells 3 times with PBS, and the number of colonies was counted using ImageJ software (version 1.8.0; Bethesda, MD, USA).

### 4.7. Cell Apoptosis Assay

HT-29 cells were planted into 6-well microplates (5 × 10^5^ cells/well), followed by treatment with PNE (10, 20, and 40 μg/mL) and 5-FU (20 μg/mL) for 48 h. Cell morphology was observed under a microscope. Cells were digested using accutase from the annexin V–FITC/PI apoptosis kit and washed with PBS twice. Next, cells were suspended in 0.5 mL of 1 × binding buffer solution, followed by staining with 5 μL annexin V–FITC and 10 μL PI for 5 min at room temperature in darkness. A flow cytometer (Thermo Fisher Scientific; Waltham, MA, USA) was employed to detect the stained apoptotic cells.

### 4.8. Measurement of MMP

HT-29 cells were plated in 6-well microplates (2 × 10^5^ cells/well) and treated with PNE (10, 20, and 40 μg/mL) and 5-FU (20 μg/mL) for 48 h. Then, the cells were stained with 0.5 mL JC-1 staining solution (0.5 mL) at 37 °C for 20 min, washed with 1 mL JC-1 staining buffer twice, and then 2 mL medium were added. Finally, the cells were observed for fluorescence intensity using an inverted fluorescence microscope (Nikon; Tokyo, Japan).

### 4.9. Animals

Female BALB/c nude mice (6–8 weeks old) weighing 18–20 g were obtained from Chengdu Dossy Experimental Animals Co., Ltd. (Chengdu, China). The mice were housed in a pathogen-free room under controlled environmental conditions of temperature (22 ± 2 °C), humidity (50–70%), and a 12 h light–dark cycle, during which they were provided with a standard diet and water. All procedures were performed following the National Institutes of Health Guide for the Care and Use of Laboratory Animals. The study was approved on 5th July 2022 by the Laboratory Animal Ethics Committee of the State Key Laboratory of Biotherapy, Sichuan University (No: 2022070502).

### 4.10. Establishment of the Xenograft Model and Treatment

The mice were adaptively fed for 7 days. Then, they were injected subcutaneously at the right side of the neck with 5 × 10^6^ HT-29 cells to induce xenograft tumor growth. After successful modeling, they were randomly allocated into 5 groups (*n* = 5): the control group received saline intragastric administration (0.2 mL/day); the PNE treatment groups received PNE intragastric administration (25, 50, 100 mg/kg/day); and the 5-FU group received 5-FU intraperitoneal administration (20 mg/kg/2 days). During the experiments, the body weight and tumor volume (TV) of mice were recorded every 2 days. After treating them for 16 days, the mice were euthanized through an intraperitoneal injection of 100 mg/kg pentobarbital sodium, and the tumor tissues were excised and weighed quickly. TV and the inhibition rate were calculated as follows: TV = tumor length × tumor width^2^ × 0.5; inhibition rate (%) = (1-mean tumor weight of the treatment group/mean tumor weight of the control group) × 100%.

### 4.11. Hematoxylin–Eosin (HE) Staining

The tumor tissues were fixed in 4% paraformaldehyde for 24 h, paraffin-embedded, and sectioned into 4 μm-thick slices. Then, the HE staining proceeded according to standard histological procedures. Finally, the samples were examined and photographed using a microscope (Nikon; Tokyo, Japan).

### 4.12. IHC Assay

Three tumor tissues from each group were randomly selected and sectioned using the same method as for the HE staining. IHC staining was performed for p53, Bax, and Bcl-2; then microscopic analysis was carried out. The average optical density (AOD) values were calculated as the expression levels of proteins using ImageJ software.

### 4.13. WB Assay

The total proteins of the tumor tissues were extracted with RIPA lysate buffer containing 1% PMSF and 1% cocktail, and then quantified with the Bradford dye reagent. Subsequently, equal amounts of total proteins from each group were loaded and separated using SDS-PAGE (Yamay; Shanghai, China), and then transferred onto polyvinylidene fluoride membranes. The membranes were blocked using 5% skim milk at room temperature for 1 h, followed by incubation with primary antibodies (p53, Bax, Bcl-2, Bcl-xL, cleaved caspase-3, and PARP were at a dilution of 1:1000, while β-actin was 1:5000, and α-Tubulin was 1:20,000) overnight at 4 °C. Afterward, the membranes were incubated with the secondary antibodies (1:5000) at room temperature for 1 h. The protein bands were visualized using an electrochemiluminescence kit (Yamay, Shanghai, China) and quantified using ImageJ software.

### 4.14. Statistical Analysis

Data are expressed as the mean ± SD. One-way ANOVA was used to compare the differences among groups, and values of *p* < 0.05 were considered statistically significant. Statistical analysis was conducted using IBM SPSS Statistics (version 25.0; Armonk, NY, USA) and GraphPad Prism (version 8.0.2; La Jolla, CA, USA).

## 5. Conclusions

This study characterized the chemical components of PNE and first explored the in vitro and in vivo inhibitory effects, as well as the preliminary mechanisms of PNE on human colorectal cancer HT-29 cells. The results revealed that PNE contained various compounds that had been reported to have antitumor activities. The in vitro and in vivo studies showed that PNE could inhibit cell proliferation and colony formation, induce early and late cell apoptosis, reduce MMP, and inhibit tumor growth in vivo. The results of mechanism research indicated that PNE activated the p53-mediated mitochondrial pathway, while it also regulated the downstream Bcl-2 family proteins, thereby activating the caspase-3 pathway and inducing cell apoptosis. This study provides a reference for the development of natural-origin anti-CRC drugs.

## Figures and Tables

**Table 1:** Major bioactive compounds in PNE.

No.	t_R_ * (Min)	Chemical Formula	Experimental Mass (*m*/*z*) [M + H]^+^	Theoretical Mass (*m*/*z*) [M + H]^+^	Error (ppm)	MS/MS Fragment Ions	Compound Identification	Peak Area%	Biological Activity
1	4.89	C_17_H_19_NO_3_	286.1447	286.1438	3.15	201.0552, 171.0445, 143.0496, 135.0445, 112.0763, 84.0815	Piperine	23.05	Antitumor, antioxidant, cardioprotective, anti-inflammatory, neuroprotective [5]
2	10.23	C_21_H_29_NO_3_	344.2231	344.2220	3.20	314.2103, 222.1859, 135.0445, 112.0763, 84.0816	Piperolein B	3.33	Antitumor, anti-inflammatory [17]
3	4.50	C_16_H_17_NO_3_	272.1289	272.1281	2.94	201.0552, 143.0496, 137.0840, 135.0445, 98.0608, 70.0660	Piperyline	2.46	Neuroprotective [18]
4	17.86	C_22_H_43_NO	338.3427	338.3417	2.96	321.3160, 303.3051, 170.1543	Erucamide	2.19	Neuroprotective [19]
5	6.47	C_14_H_25_NO	224.2015	224.2009	2.68	168.1389, 151.1122	Pellitorine	1.34	Antitumor, anti-inflammatory [12,20]
6	8.90	C_21_H_27_NO_3_	342.2074	342.2064	2.92	135.0445	Propafenone	1.23	Cardioprotective [21]
7	4.83	C_17_H_21_NO_3_	288.1602	288.1594	2.78	175.0756, 138.0918, 135.0445, 112.0764	Piperanine	1.22	Neuroprotective [22]
8	13.11	C_24_H_33_NO_3_	384.2543	384.2533	2.60	283.1703, 161.0602, 135.0445	Guineensine	1.06	Neuroprotective, anti-inflammatory [23]
9	3.44	C_18_H_19_NO_4_	314.1395	314.1387	2.55	177.0551, 149.0599, 145.0288, 121.0653	N-trans-Feruloyltyramine	0.43	Antitumor, antioxidant, anti-inflammatory [24]
10	0.69	C_7_H_7_NO_2_	138.0554	138.0550	2.90	94.0658	Trigonelline	0.20	Antitumor, cardioprotective, neuroprotective [25]
11	0.69	C_5_H_11_NO_2_	118.0868	118.0863	4.23	101.0601	Betaine	0.08	Antitumor, antioxidant, anti-inflammatory, neuroprotective [26]
12	15.07	C_18_H_35_NO	282.2798	282.2791	2.48	265.2531, 247.2425	Oleamide	0.05	Antitumor, neuroprotective [27,28]
13	4.57	C_16_H_21_NO_3_	276.1604	276.1594	3.62	175.0760, 135.0445	4, 5-Dihydropiperlonguminine	0.01	Antitumor, neuroprotective [29,30]

* t_R_, retention time.

## Data Availability

Data are contained within article and Supplementary Materials.

## Supplementary Materials

The following supporting information can be downloaded at: https://www.mdpi.com/article/10.3390/ph16091325/s1, Figure S1: Total ion current (TIC) of the main pharmacologically active compounds in PNE; Table S1: Identification results of chemical components in PNE.

## Abbreviations

5-FU	5-fluorouracil
AOD	Average optical density
CRC	Colorectal cancer
HE	Hematoxylin–eosin
IHC	Immunohistochemistry
MMP	Mitochondrial membrane potential
OD	Optical density
PNE	*Piper nigrum* extract
TV	Tumor volume
WB	Western blot
