# Supplementary material for: Piper nigrum Extract Inhibits the Growth of Human Colorectal Cancer HT-29 Cells by Inducing p53-Mediated Apoptosis
Source: Pharmaceuticals (Basel). 2023 Sep 19;16(9):1325. doi: 10.3390/ph16091325 (PMC10537627; doi:10.3390/ph16091325)
Supplement: Supplementary file 1 [file pharmaceuticals-16-01325-s001.zip › pharmaceuticals-2584801-supplementary.pdf]

pharmaceuticals

Supplementary Materials: *Piper nigrum* extract inhibits the growth of human colorectal cancer HT-29 cells by inducing p53-mediated apoptosis

Rui Wu [1], Jiajia Zhao [1], Panhong Wei [1], Minghai Tang [2], Ziyan Ma [2], Yunyan Zhao [1], Leilei Du [1] and Li Wan [1,*]

[1] State Key Laboratory of Southwestern Chinese Medicine Resources, School of Pharmacy, Chengdu University of Traditional Chinese Medicine, Chengdu 611137, China; wurui2298@163.com (R.W.); zjj11242020@163.com (J.Z.); wph0816@163.com (P.W.); yunyan_zhao2022@163.com (Y.Z.); duleilei@cdutcm.edu.cn (L.D.)

[2] State Key Laboratory of Biotherapy and Cancer Center, National Clinical Research Center for Geriatrics, West China Hospital of Sichuan University, Chengdu 610041, China; tangminghai123@sina.cn (M.T.); maziyan@stu.scu.edu.cn (Z.M.)

* Correspondence: wanli@cdutcm.edu.cn; Tel.: +86-15982418713

Table S1. Identification results of chemical components in PNE.

No.	t_R (min)	Compound	Chemical formula	Type	Peak area %
1	4.89	Piperine	$C_{17}H_{19}NO_3$	Alkaloids	23.0535
2	10.23	Piperolein B	$C_{21}H_{29}NO_3$	Alkaloids	3.3270
3	6.47	Gravelliferone	$C_{19}H_{22}O_3$	Coumarins	2.6384
4	4.50	Piperyline	$C_{16}H_{17}NO_3$	Alkaloids	2.4596
5	17.86	Erucamide	$C_{22}H_{43}NO$	Alkaloids	2.1805
6	7.48	Diisobutyl phthalate	$C_{16}H_{22}O_4$	Esters	1.7972
7	15.67	(2E,4E,12Z)-N-(2-methylpropyl)octadeca-2,4,12-trienamide	$C_{22}H_{39}NO$	Alkaloids	1.7744
8	16.54	Stearoylglycerol	$C_{21}H_{42}O_4$	Esters	1.6109
9	6.47	Pellitorine	$C_{14}H_{25}NO$	Alkaloids	1.3397
10	8.90	Propafenone	$C_{21}H_{27}NO_3$	Phenylpropanoid	1.2255
11	4.83	Piperanine	$C_{17}H_{21}NO_3$	Alkaloids	1.2184
12	13.11	Guineensine	$C_{24}H_{33}NO_3$	Alkaloids	1.0599
13	5.25	Bis(4-ethylbenzylidene)sorbitol	$C_{24}H_{30}O_6$	Alcohols	0.6262
14	3.44	N-trans-Feruloyltyramine	$C_{18}H_{19}NO_4$	Alkaloids	0.4289
15	10.41	(2E,4E)-N-(2-methylpropyl)dodeca-2,4-dienamide	$C_{16}H_{29}NO$	Alkaloids	0.2147
16	3.36	Dipropylene glycol dimethyl ether	$C_8H_{18}O_3$	Other	0.2102
17	0.69	Trigonelline	$C_7H_7NO_2$	Alkaloids	0.2016
18	15.27	Oleoyl ethanolamide	$C_{20}H_{39}NO_2$	Alkaloids	0.1710
19	16.37	Dioctyl phthalate	$C_{24}H_{38}O_4$	Esters	0.1708
20	13.44	Dibutyl sebacate	$C_{18}H_{34}O_4$	Esters	0.1629
21	14.91	brachystamide B	$C_{26}H_{37}NO_3$	Alkaloids	0.1214
22	0.67	Asparagine	$C_4H_8N_2O_3$	Amino acids	0.1211
23	4.35	3-[(2H-1,3-benzodioxol-5-yl)methyl]-4-[(3,4-dimethoxyphenyl)methyl]oxolan-2-one	$C_{21}H_{22}O_6$	Lignans	0.1137
24	13.93	(2S)-2-[(2R)-7-(2-Methoxyethoxy)-5,8-dimethyl-1,2,3,4-tetrahydro-2-naphthalenyl]-1-(1-piperidinyl)-1-propanone	$C_{23}H_{35}NO_3$	Alkaloids	0.1113
25	3.75	Triethylcitrate	$C_{12}H_{20}O_7$	Esters	0.1007
26	0.78	L-Malicacid	$C_4H_6O_5$	Organic acids	0.0958
27	0.66	Choline	$C_5H_{13}NO$	Alkaloids	0.0859
28	0.69	Betaine	$C_5H_{11}NO_2$	Alkaloids	0.0786

29	2.46	N-Methylhernagine	$C_{20}H_{23}NO_4$	Alkaloids	0.0777
30	0.69	Gluconicacid	$C_6H_{12}O_7$	Organic acids	0.0762
31	14.74	Bis(2-ethylhexyl)phthalate	$C_{24}H_{38}O_4$	Esters	0.0752
32	7.41	Trilostane	$C_{20}H_{27}NO_3$	Steroids	0.0581
33	16.48	Bis(2-ethylhexyl)adipate	$C_{22}H_{42}O_4$	Esters	0.0549
34	16.19	PheophorbideA	$C_{35}H_{36}N_4O_5$	Other	0.0488
35	15.07	Oleamide	$C_{18}H_{35}NO$	Alkaloids	0.0466
36	0.74	Adenine	$C_5H_5N_5$	Nucleosides	0.0383
37	0.71	D-(-)-Fructose	$C_6H_{12}O_6$	Saccharides	0.0381
38	0.90	Acetophenone	C_8H_8O	Other	0.0350
39	2.11	decaffeoylverbascoside	$C_{20}H_{30}O_{12}$	Glycosides	0.0261
40	14.61	Hexadecanamide	$C_{16}H_{33}NO$	Alkaloids	0.0253
41	5.39	(3aR,4aS,5R,6S,8S,9aR)-5,6-dihydroxy-4a,8-dimethyl-3-methylenedecahydroazuleno[6,5-b]furan-2(3H)-one	$C_{15}H_{22}O_4$	Esters	0.0247
42	4.02	N-Butylbenzenesulfonamide	$C_{10}H_{15}NO_2S$	Alkaloids	0.0230
43	11.16	2-Amino-1,3,4-octadecanetriol	$C_{18}H_{39}NO_3$	Alcohols	0.0219
44	0.90	4-Oxoproline	$C_5H_7NO_3$	Amino acids	0.0213
45	1.27	DL-Norleucine	$C_6H_{13}NO_2$	Amino acids	0.0188
46	0.89	L-Pyroglutamicacid	$C_5H_7NO_3$	Amino acids	0.0185
47	3.34	BetulalbusideA	$C_{16}H_{28}O_7$	Glycosides	0.0168
48	14.79	(2E,4E,9Z)-1-(piperidin-1-yl)hexadeca-2,4,9-trien-1-one	$C_{21}H_{35}NO$	Alkaloids	0.0155
49	0.68	4-Aminobutyricacid	$C_4H_9NO_2$	Amino acids	0.0154
50	6.33	3,5-di-tert-Butyl-4-hydroxybenzaldehyde	$C_{15}H_{22}O_2$	Phenols	0.0153
51	4.66	Benzophenone	$C_{13}H_{10}O$	Other	0.0144
52	3.61	Azelaicacid	$C_9H_{16}O_4$	Organic acids	0.0130
53	0.90	Guanosine	$C_{10}H_{13}N_5O_5$	Nucleosides	0.0127
54	8.28	Dodecylsulfate	$C_{12}H_{26}O_4S$	Organic acids	0.0125
55	0.90	N-Acetyl-DL-glutamicacid	$C_7H_{11}NO_5$	Amino acids	0.0124
56	5.62	methyl3-((1E,3E)-3,5-dimethylhepta-1,3-dien-1-yl)-8-hydroxy-6a,8-dimethyl-6-oxo-6a,8,9,9a-tetrahydro-6H-furo[2,3-h]isochromene-9-carboxylate	$C_{24}H_{30}O_6$	Other	0.0117
57	4.22	Monobutylphthalate	$C_{12}H_{14}O_4$	Esters	0.0116
58	0.68	L-Asparticacid	$C_4H_7NO_4$	Amino acids	0.0109
59	4.98	6-Gingerol	$C_{17}H_{26}O_4$	Phenols	0.0107
60	2.58	Isophthalicacid	$C_8H_6O_4$	Organic acids	0.0107
61	1.69	2-Phenylglycine	$C_8H_9NO_2$	Amino acids	0.0106
62	0.75	Guanine	$C_5H_5N_5O$	Nucleosides	0.0104
63	0.71	D-Proline	$C_5H_9NO_2$	Amino acids	0.0103
64	12.12	tetradecylhydrogensulphate	$C_{14}H_{30}O_4S$	Organic acids	0.0094
65	2.82	4-Nitrophenol	$C_6H_5NO_3$	Phenols	0.0089
66	2.11	[(2R,3S,4S,5R,6R)-3,4,5-trihydroxy-6-[2-(3-hydroxy-5-oxooxolan-3-yl)propoxy]oxan-2-yl]methyl(2E)-3-(3,4-dihydroxyphenyl)prop-2-enoate	$C_{22}H_{28}O_{12}$	Glycosides	0.0084
67	4.57	4,5-Dihydropiperlonguminine	$C_{16}H_{21}NO_3$	Alkaloids	0.0084
68	3.42	D-Panthenol	$C_9H_{19}NO_4$	Alcohols	0.0084
69	1.81	Pantothenicacid	$C_9H_{17}NO_5$	Organic acids	0.0081
70	1.72	L-Phenylalanine	$C_9H_{11}NO_2$	Amino acids	0.0078
71	0.78	Pipecolicacid	$C_6H_{11}NO_2$	Amino acids	0.0077
72	2.96	Eriocitrin	$C_{27}H_{32}O_{15}$	Glycosides	0.0067
73	0.95	Succinicacid	$C_4H_6O_4$	Organic acids	0.0066
74	12.26	4-Dodecylbenzenesulfonicacid	$C_{18}H_{30}O_3S$	Organic acids	0.0065

75	2.17	Indole-3-acrylicacid	$C_{11}H_9NO_2$	Organic acids	0.0063
76	0.88	Uridine	$C_9H_{12}N_2O_6$	Nucleosides	0.0058
77	10.66	5-OxoETE	$C_{20}H_{30}O_3$	Organic acids	0.0056
78	6.99	Mono(2-ethylhexyl)phthalate	$C_{16}H_{22}O_4$	Organic acids	0.0047
79	0.92	D-(+)-Malicacid	$C_4H_6O_5$	Organic acids	0.0044
80	10.66	12,13-dihydroxy-9-octadecenoicacid	$C_{18}H_{34}O_4$	Organic acids	0.0039
81	3.68	4-Methoxycinnamaldehyde	$C_{10}H_{10}O_2$	Phenylpropanoid	0.0035
82	2.58	2-(3,4-Dihydroxyphenyl)-5-hydroxy-4-oxo-4H-chromen-7-yl6-O-(6-deoxy-alpha-L-mannopyranosyl)-beta-D-glucopyranoside	$C_{27}H_{30}O_{15}$	Glycosides	0.0034
83	1.66	3-Hydroxymandelicacid	$C_8H_8O_4$	Organic acids	0.0034
84	1.57	Salsoline	$C_{11}H_{15}NO_2$	Alkaloids	0.0031
85	0.88	L-citramalicacid	$C_5H_8O_5$	Organic acids	0.0030
86	1.91	Protocatechuicacid	$C_7H_6O_4$	Phenols	0.0025
87	16.31	Elaidicacid	$C_{18}H_{34}O_2$	Organic acids	0.0025
88	2.79	N-Acetyl-D-alloisoleucine	$C_8H_{15}NO_3$	Amino acids	0.0022
89	4.39	(+)-trans-C75	$C_{14}H_{22}O_4$	Esters	0.0021
90	3.40	9-(2,3-dihydroxypropoxy)-9-oxononanoicacid	$C_{12}H_{22}O_6$	Organic acids	0.0020
91	1.66	Gentisicacid5-O-β-D-glucopyranoside	$C_{13}H_{16}O_9$	Glycosides	0.0020
92	2.27	osmanthusideH	$C_{19}H_{28}O_{11}$	Glycosides	0.0019
93	15.37	17-Octadecynoicacid	$C_{18}H_{32}O_2$	Organic acids	0.0018
94	1.89	Vanillylalcohol	$C_8H_{10}O_3$	Phenols	0.0017
95	14.42	16-Hydroxyhexadecanoicacid	$C_{16}H_{32}O_3$	Organic acids	0.0016
96	6.04	perfluorooctane-1-sulfonicacid	$C_8HF_{17}O_3S$	Organic acids	0.0010
97	4.68	Dodecanedioicacid	$C_{12}H_{22}O_4$	Organic acids	0.0008

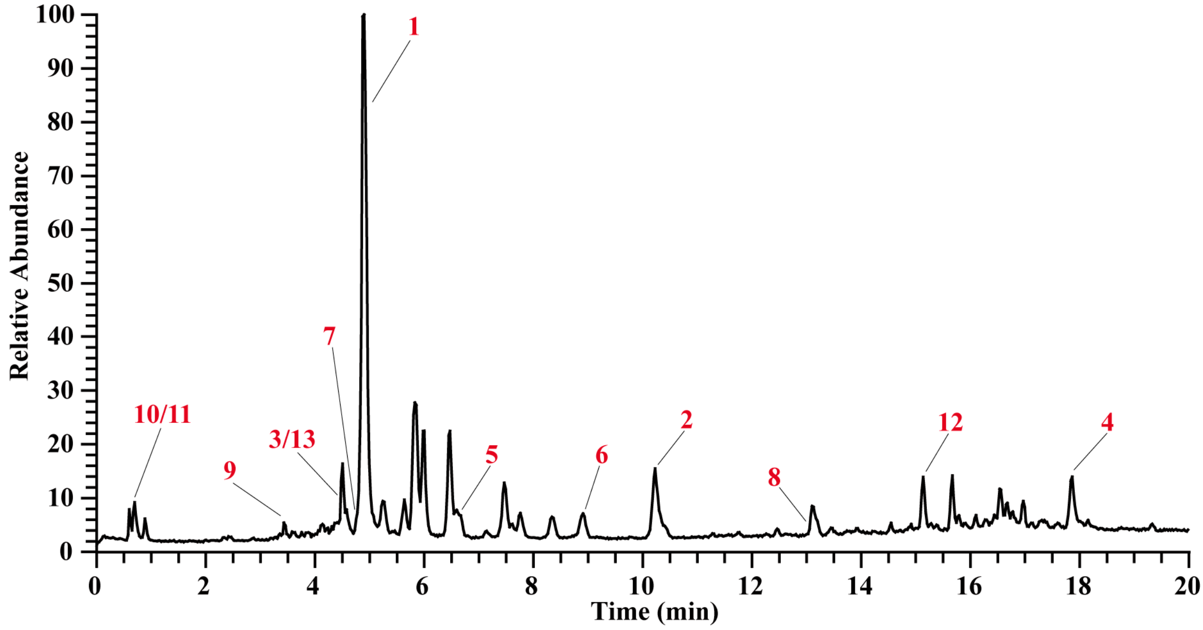

Figure S1. Total ions current (TIC) of the main pharmacological active compounds in PNE.
